# Computational Reproducibility of Molecular Phylogenies

**DOI:** 10.1093/molbev/msad165

**Published:** 2023-07-19

**Authors:** Sudhir Kumar, Qiqing Tao, Alessandra P Lamarca, Koichiro Tamura

**Affiliations:** Institute for Genomics and Evolutionary Medicine, Temple University, Philadelphia, PA, USA; Department of Biology, Temple University, Philadelphia, PA, USA; Institute for Genomics and Evolutionary Medicine, Temple University, Philadelphia, PA, USA; Department of Biology, Temple University, Philadelphia, PA, USA; Institute for Genomics and Evolutionary Medicine, Temple University, Philadelphia, PA, USA; Department of Biology, Temple University, Philadelphia, PA, USA; Department of Genetics, Federal University of Rio de Janeiro, Rio de Janeiro, Brazil; Research Center for Genomics and Bioinformatics, Tokyo Metropolitan University, Hachioji, Tokyo, Japan; Department of Biological Sciences, Tokyo Metropolitan University, Hachioji, Tokyo, Japan

**Keywords:** molecular phylogenies, reproducibility, maximum likelihood, optimality

## Abstract

Repeated runs of the same program can generate different molecular phylogenies from identical data sets under the same analytical conditions. This lack of reproducibility of inferred phylogenies casts a long shadow on downstream research employing these phylogenies in areas such as comparative genomics, systematics, and functional biology. We have assessed the relative accuracies and log-likelihoods of alternative phylogenies generated for computer-simulated and empirical data sets. Our findings indicate that these alternative phylogenies reconstruct evolutionary relationships with comparable accuracy. They also have similar log-likelihoods that are not inferior to the log-likelihoods of the true tree. We determined that the direct relationship between irreproducibility and inaccuracy is due to their common dependence on the amount of phylogenetic information in the data. While computational reproducibility can be enhanced through more extensive heuristic searches for the maximum likelihood tree, this does not lead to higher accuracy. We conclude that computational irreproducibility plays a minor role in molecular phylogenetics.

## Introduction

In computational sciences, irreproducibility is observed when the same program, executed multiple times, yields disparate results under identical analytical conditions (Sonnenburg et al. 2007; Rougier et al. 2017). This phenomenon, termed computational irreproducibility, is distinct from general irreproducibility of results, which arises due to changes in models, methods, algorithms, and data sets leading to varying outcomes (Som 2014; Ritchie et al. 2017; Shen et al. 2017). Conventionally, in the field of molecular phylogenetics, it has been expected that the execution of the same program, utilizing the same data set and applying the same models and assumptions, will produce the same phylogeny. That is, the results will be computationally reproducible. However, the lack of computational reproducibility has been reported in many scientific disciplines (Magee et al. 2014; Marjanović and Laurin 2018; Zhou et al. 2018; Salomaki et al. 2020; Shen et al. 2020; Young and Gillung 2020).

In molecular phylogenetics, Shen et al. (2020) systematically analyzed computational reproducibility in the inference of phylogenies using the maximum likelihood (ML) method. They compared the phylogenies generated by executing the same program twice on identical data sets, utilizing the same substitution model and heuristic search parameters. The only variation was the random seed used in the two heuristic searches for the ML tree. Their analyses found that 9–18% of the inferences led to divergent phylogenies. On average, these data sets contained less phylogenetic information compared to those yielding reproducible phylogenies (Shen et al. 2020). Furthermore, the irreproducible phylogenies were less accurate in reconstructing the true tree. These patterns of irreproducibility, especially their correlation with phylogenetic inaccuracies, are a matter of concern. Consequently, a deeper understanding of the causes and effects of computational irreproducibility in inferred phylogenies and their accuracy is imperative.

From an evolutionary perspective, irreproducibility becomes a matter of significant concern if a single program run generates a phylogeny that reconstructs evolutionary relationships with less accuracy than another run of the same program. Concerns also arise if the irreproducibility is linked to the low optimality score of the inferred phylogeny, implying that the topological space explored in the initial run was insufficient and a potentially more accurate phylogeny with higher log-likelihood remained undiscovered. Despite reports of the computational reproducibility of phylogenies (Zhou et al. 2018; Shen et al. 2020), these fundamental questions remain unresolved. If these concerns are validated, irreproducibility in molecular phylogenetics could impede the development of general biological patterns, delay scientific consensus, and mislead future evolutionary investigations.

Hence, our study aimed to compare the accuracies and log-likelihoods of alternative phylogenies for both computer-simulated and empirical data sets that suffered from phylogeny irreproducibility. Alongside, we investigated fundamental causes for the observed irreproducibility patterns, their connection with the accuracy of inferred phylogenies, and their respective optimality scores.

## Results and Discussion

**Fig. 1.**
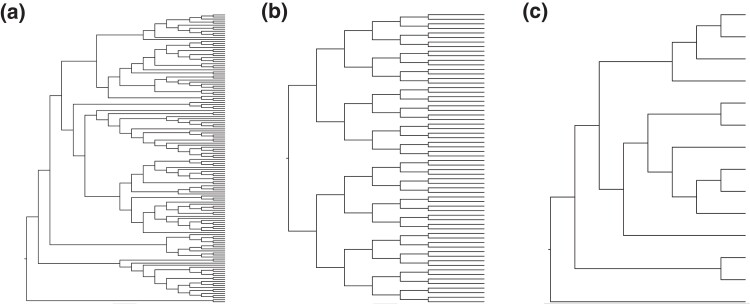
Topologies utilized as the true tree in reproducibility analysis. (*a*) Phylogeny of 142 mammalian taxa used to generate the 100-data set collection of simulated alignments. (*b*) The tree used for simulating the 7,500-data set collection. (*c*) The multispecies coalescence tree of a subset of species (14) inferred using Chen et al. (2019) data set to ensure 100% bootstrap support and Bayesian posterior probabilities of 1.0. This was used as the reference tree for the 1,000-genes collection.

Our approach involved comparing phylogenies generated in separate runs of the same program, both with each other and the known (correct) tree. We conducted two-run ML analyses of computer-simulated alignments of 142 species, originally generated using a model tree (fig. 1*a*) and empirically determined evolutionary parameters. These parameters included a wide range of evolutionary rates (0.81–3.95 × 10^−9^ substitutions per site per year), base composition biases (39–82% G + C content), and transition/transversion rate ratios (1.35–2.6). From this collection, we selected 100 alignments at random for analysis using IQ-TREE 2.1.3 (Minh et al. 2020) and RaxML-NG 1.1.0 (Kozlov et al. 2019). Furthermore, we re-analyzed phylogenies generated by IQ-TREE 2 analysis of 7,500 alignments (Shen et al. 2020). This allowed us to test the generality of patterns observed for the 100-data set collection. The sequence alignments in the 7,500-data set collection were also simulated using a wide range of informativeness and sequence lengths for the phylogeny depicted in figure 1*b*.

In addition to the computer-simulated data sets, we analyzed an empirical data set of gene alignments compiled by Chen et al. (2019). Given that the true tree is unknown for empirical data sets, we utilized a pruned version of their multispecies coalescence phylogeny in Chen et al. (2019) as the reference tree to ensure that all the inferred clades had 100% posterior probability and bootstrap support values (fig. 1*c*). We selected sequence alignments of 1,000 genes for IQ-TREE 2 analysis.

### Relative Accuracies of Irreproducible Phylogenies

**Fig. 2.**
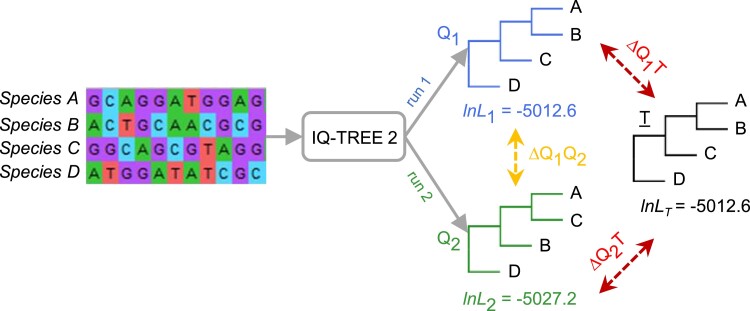
An analysis of computational reproducibility in phylogenetics for the 100-data set collection. Two runs (1 and 2) of the same program using the same sequence alignment and substitution models may not produce the same tree (e.g., *Q*_1_ ≠ *Q*_2_ for IQ-TREE 2), resulting in phylogeny irreproducibility (Δ*Q*_1_*Q*_2_; vertical yellow arrow). Red arrows mark comparisons between the inferred trees and the true tree (*T*).

**Fig. 3.**
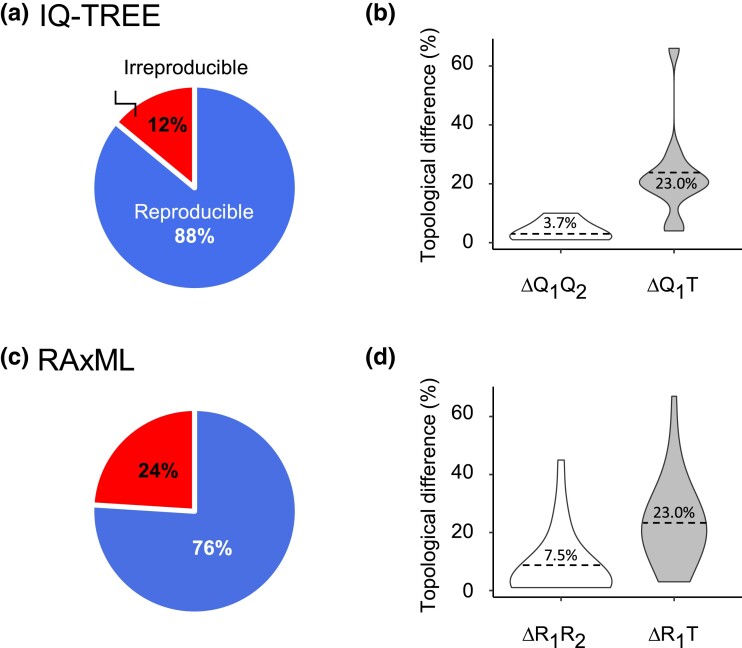
Frequency of irreproducible phylogenies and their accuracy in the 100-data set collection. Percentage of simulated alignments for which identical and different trees were produced in two runs of (*a*) IQ-TREE 2 and (*c*) RAxML. The violin plots show the distribution of topological differences between the first- and second-run trees (white, irreproducibility) and first-run and the true tree (gray, accuracy) for (*b*) IQ-TREE 2 and (*d*) RAxML. The *X*-axis of violin plots corresponds to the density of observations, with wider parts of the violin corresponding to a higher density of values. The dotted lines correspond to the average values. “1,” “2,” “*Q*,” “*R*,” and “*T*” denote the first run, second run, IQ-TREE 2, RAxML, and the true tree, respectively.

We executed IQ-TREE 2 twice using identical hardware, parameters, and heuristic search conditions (except for the random seed, see Materials and Methods) for each alignment in the 100-data set collection (fig. 2). There were 12 instances in which the second-run phylogenies (*Q*_2_) were different from the first-run phylogenies (*Q*_1_) (fig. 3*a*). These findings reaffirmed the presence of significant computational irreproducibility previously reported by Shen et al. (2020). For these irreproducible phylogenies, more than 23% of the evolutionary relationships in *Q*_1_ differed from the true tree (*T*; mean Δ*Q*_1_*T* = 23.6%). Intriguingly, the same amount of phylogenetic inaccuracy was observed in the second-run phylogenies. This inaccuracy exceeded the difference between *Q*_1_ and *Q*_2_ (mean Δ*Q*_1_*Q*_2_ = 3.7%). Meaning, on average, Δ*Q*_1_*Q*_2_ was less than Δ*Q*_1_*T* and Δ*Q*_2_*T* (white vs. gray violin plots in fig. 3*b*). Comparable trends were found in the RAxML analysis of the same data set collection (fig. 3*c* and *d*), as the first- and the second-run phylogenies (*R*_1_ and *R*_2_) had a similar degree of phylogenetic error (mean inaccuracies = 23.1% and 23.0%, respectively). But they were much more similar to each other (mean Δ*R*_1_*R*_2_ = 7.5%).

**Fig. 4.**
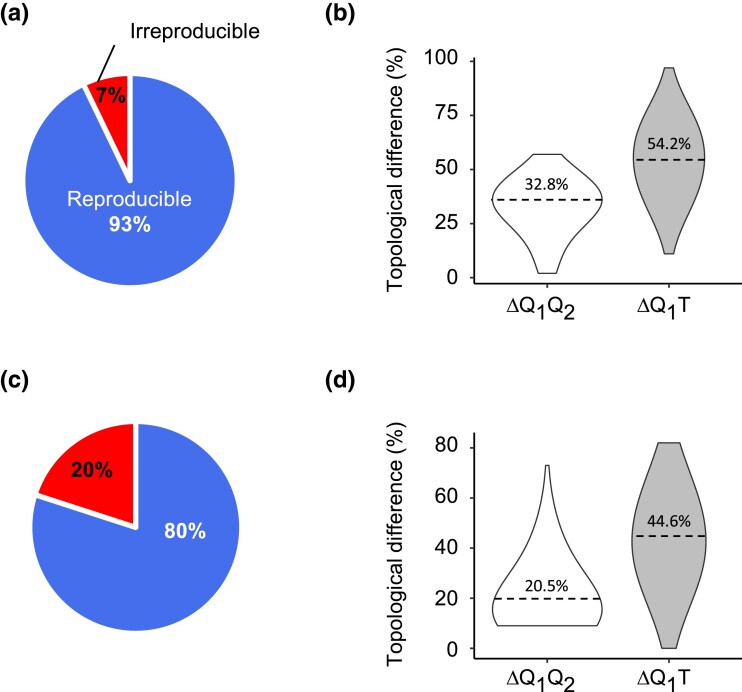
Reproducibility results for the IQ-TREE 2 analysis of the 7,500-data set and the empirical 1,000-gene collections. Pie charts show the proportions of data sets producing the same (reproducible) and different (irreproducible) phylogenies in two runs of IQ-TREE 2 for (*a*) 7,500-alignment data set and (*c*) empirical 1,000-gene data set. The violin plots show the distributions of topological differences between the first- and second-run phylogenies (white violins) and between the first-run and the true tree (gray violins) for (*b*) 7,500-alignment data set and (*d*) the empirical 1,000-gene data set. The *X*-axis of violin plots corresponds to the density of observations, with wider parts of the violin corresponding to higher density of values. Dashed lines show the mean values of the distributions. “1,” “2,” “*Q*,” and “*T*” denote the first run, second run, IQ-TREE 2, and the true tree, respectively.

An analysis of the 7,500-data set collection confirmed the patterns observed in the 100-data set collection (fig. 4*a* and *b*). Irreproducibility was found in 7.2% of the alignments, and the alternative phylogenies generated exhibited equivalent inaccuracies (54.3% and 54.1%). Given that the two data collections were simulated under different conditions yet produced similar trends, we anticipate these trends to be found for other tree topologies, sequence lengths, and substitution patterns. Indeed, a 1,000-gene collection of empirical data sets produced concordant patterns (fig. 4*c* and *d*). Analysis of 20.5% of the genes resulted in irreproducible phylogenies, and the first- and second-run phylogenies differed equally from the reference tree (mean difference of 43.6% and 43.8%). Once again, the difference between the first- and second-run phylogenies was considerably smaller (mean = 20.5%) than the inaccuracy of the phylogeny (fig. 4*d*). Therefore, the two runs did not produce phylogenies with significantly different levels of accuracy.

**Fig. 5.**
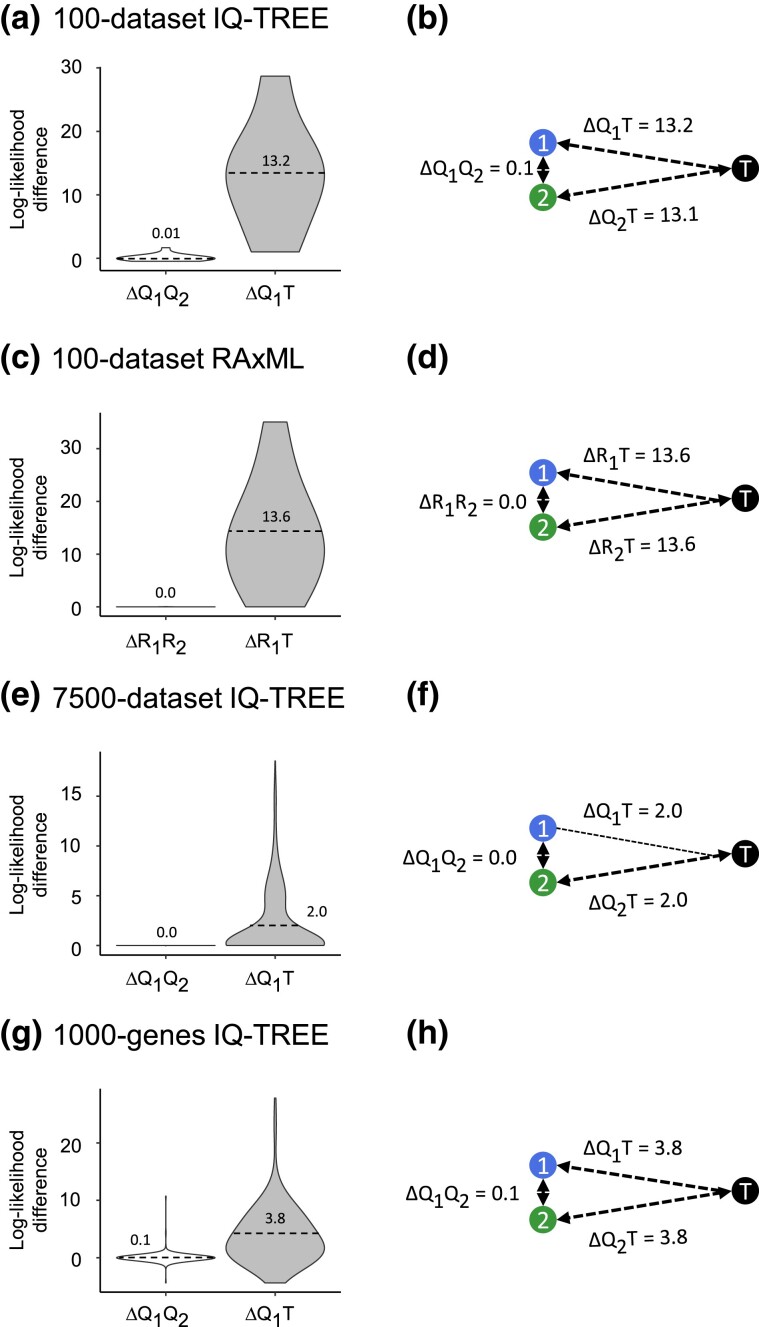
A comparison of optimality scores of irreproducible phylogenies for three data collections. Panels on the left contain violin plots showing the distributions of differences in log-likelihoods between the first- and the second-run phylogenies (white) and between the first-run phylogeny and the true tree (gray) for alignments producing irreproducible phylogenies for various combinations of data collections and inference methods (*a*, *c*, *e*, and *g*). The *X*-axis of violin plots shows the density of observations, with wider parts of the violin corresponding to a higher density of values. A positive difference means a higher likelihood for the first-run phylogeny. Panels on the right show the average of absolute log-likelihood differences between phylogenies inferred in two runs of the software and these phylogenies’ differences from the true tree (*b*, *d*, *f*, and *h*). “1,” “2,” “*Q*,” “*R*,” and “*T*” denote the first run, second run, IQ-TREE 2, RAxML, and the true tree, respectively.

The observed differences in the statistical qualities of irreproducible phylogenies are even less significant because the first-run phylogenies already boasted superior log-likelihoods compared to the true tree (fig. 5*a* and *c*). This pattern aligns with previous studies that showed inferred phylogenies to have optimality scores superior to that of the true tree (Nei et al. 1998). Notably, the highest log-likelihood difference between the true and inferred tree was 35.7 for the 100-alignment data set and 142.6 for the empirical 1,000-genes collection, which is quite large. Also, alternative trees tended to have similar log-likelihoods (fig. 5*b* and *d*). These patterns confirm that the difference between the alternate phylogenies is generally smaller than their difference from the reference/true tree. Thus, computationally irreproducible phylogenies are substantially less different from one another than they are from the true tree in terms of topological accuracy and optimality scores.

### Lack of Phylogeny Reproducibility and the Extent of the Heuristic Search

**Fig. 6.**
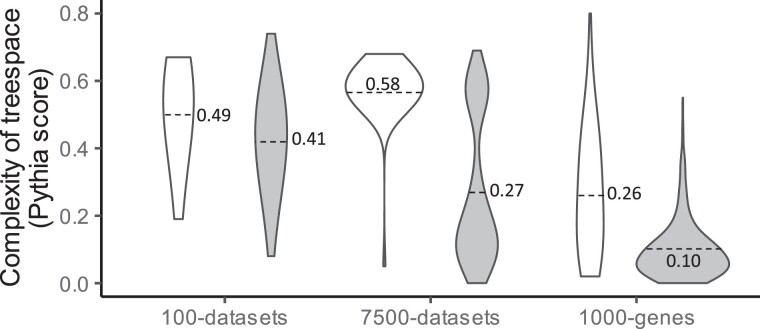
Violin plots showing the distributions of Pythia scores (treespace complexity) of different data set collections that resulted in irreproducible (white) and reproducible (gray) phylogenies. The *X*-axis of violin plots corresponds to the density of observations, with wider parts of the violin showing a higher density of values. A dotted line marks the average Pythia score for each data collection.

In the aforementioned investigation and Shen et al. (2020), all the alignments in the data collections were subjected to heuristic searches under the same set of parameters. However, it is well appreciated that some alignments require more extensive heuristic searches than others. Accordingly, numerous options are available in various software to optimize heuristic searches (Kozlov et al. 2019; Minh et al. 2020; Tamura et al. 2021). Haag et al. (2022) have developed a metric, implemented in Pythia software, to quantify the complexity of heuristic searches related to the presence of many local optima (Sanderson et al. 2011; John 2017). Alignments receive a score ranging from 0 to 1, with higher scores suggesting that the given alignment may require more extensive tree searching to reach the ML tree. We found the distribution of Pythia scores for 100-data sets collection to be quite broad (fig. 6). The alignments exhibiting phylogeny irreproducibility had a higher average score (0.51), indicating that they needed a more extensive heuristic search than the alignments with reproducible phylogenies (0.43). The difference was substantially larger for the 7,500-data set and empirical 1,000-gene collections (fig. 6).

Therefore, an ideal study investigating the reproducibility of phylogenies should conduct heuristic searches that are responsive to the complexity of the tree space searched, ensuring a similar probability of finding the ML tree across data sets. However, this is currently not feasible as determining the optimal number of heuristic searches and the scope of tree searching remains challenging (Haag et al. 2022). To test the hypothesis that expanding the heuristic search to include the island of trees containing the true tree would enhance the accuracy of the inferred phylogenies, we devised an experiment in which the topology of the true tree was supplied as the initial tree to the heuristic search in IQ-TREE 2 analysis. This guaranteed thorough exploration of the topological neighborhood of the true tree in the ML tree search. We then compared the topology with the highest likelihood produced by this analysis (*Q*_3_) with the true tree (*T*) to test the hypothesis that a more accurate phylogeny will be inferred if the heuristic search reached and evaluated phylogenies in the island that includes the true tree.

**Fig. 7.**
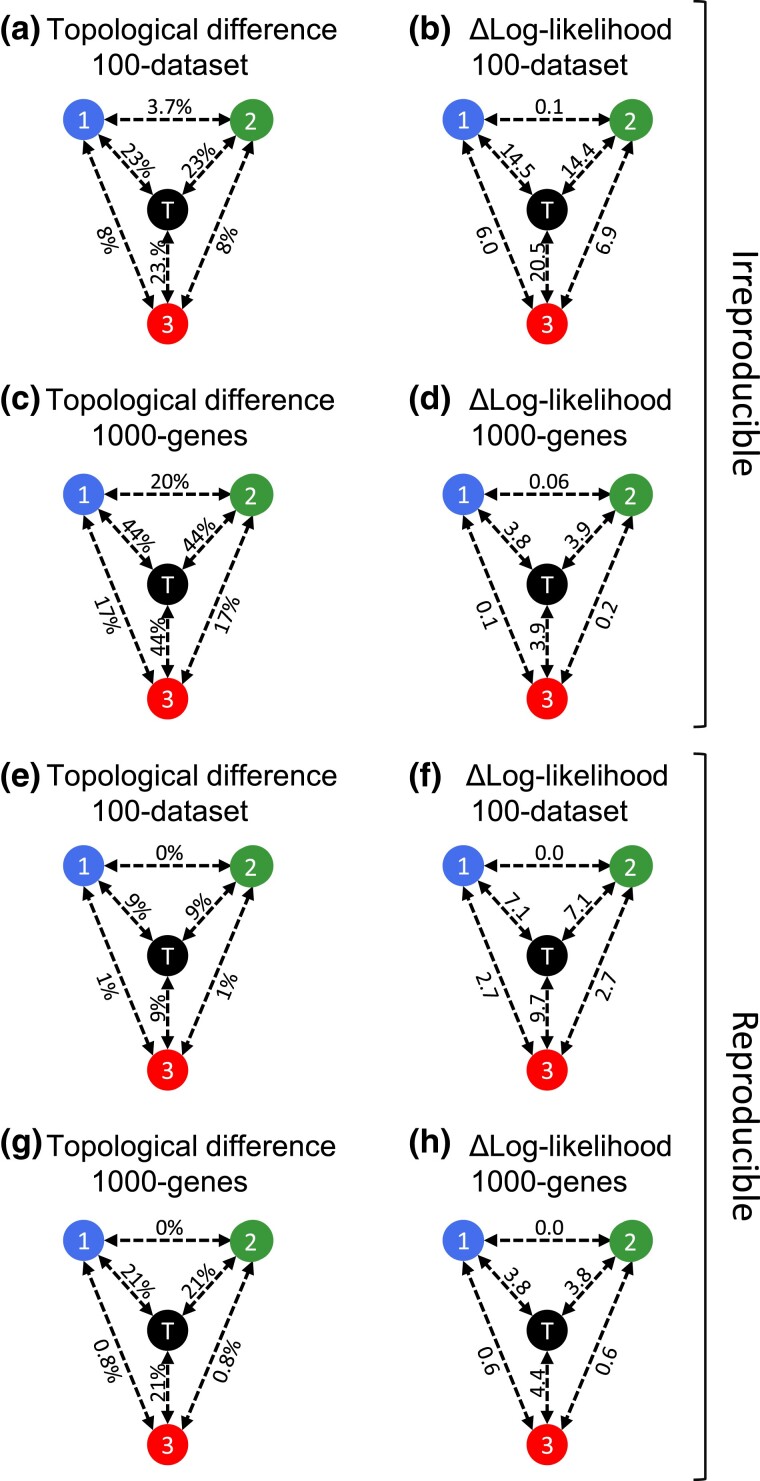
Topological and log-likelihood differences between the first-run, second-run, and true tree for data sets resulting in irreproducible phylogenies. The average percent topological difference between *Q*_1_, *Q*_2_, *Q*_3_, and the true tree (*T*) is shown for (*a*) 100-data set collection and (*c*) 1,000-genes collection. The differences between log-likelihoods of *Q*_1_, *Q*_2_, *Q*_3_, and *T* are shown for (*b*) 100-data set collection and (*d*) 1,000-genes collection. In *e*–*h*, the mean topological and log-likelihood differences are shown between the reproducible trees for both data set collections.

Intriguingly, the inaccuracies of the *Q*_3_ phylogenies were similar to those of *Q*_1_ and *Q*_2_ (fig. 7*a* and *c*). This similarity in the accuracy was not due to the identity of *Q*_3_ with *Q*_1_ or *Q*_2_, as the topological differences between *Q*_1_, *Q*_2,_ and *Q*_3_ were similar. However, the average log-likelihoods of *Q*_3_ were higher than *Q*_1_ and *Q*_2_ (fig. 7*b* and *d*). Hence, discovering phylogenies with higher log-likelihoods did not improve the phylogeny accuracy for data sets exhibiting irreproducibility. We observed analogous trends for data sets with reproducible phylogenies (fig. 7*e*–*h*).

### Forest of Trees With High Log-Likelihoods

**Fig. 8.**
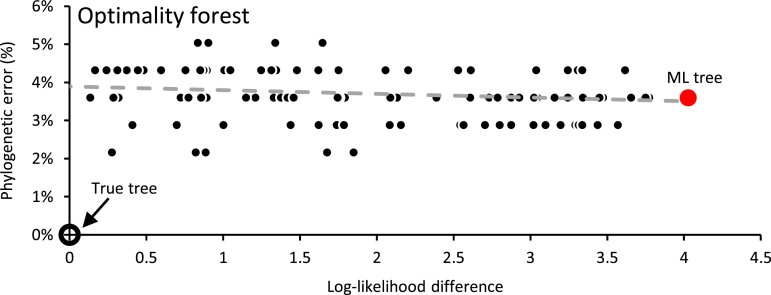
The forest of phylogenies with log-likelihoods higher than that for the true tree for an alignment of 142 species and 9,359 bases. This optimality forest contains 110 distinct phylogenies (black dots) with a higher ML than the true tree (the open black circle at the bottom left). The gray dashed line represents the linear regression line. The large red circle is for the phylogeny with the highest log-likelihood.

To gain a deeper insight into the ensemble of trees with likelihoods superior to the true tree (termed the “optimality forest”), we conducted heuristic searches using various initial trees and random seeds for a representative alignment from the 100-data set collection. The log-likelihoods and inaccuracies of the discovered and explored phylogenies are depicted in figure 8. This graph contains horizontal and vertical bands. Horizontal bands show phylogenies with the same inaccuracies but exhibit different log-likelihoods, whereas vertical bands comprise phylogenies with similar log-likelihoods yet varying degrees of inaccuracies. Notably, there is no significant correlation between the log-likelihood difference and phylogenetic error within the optimality forest, as suggested by a flat regression line (represented by the gray dashed line). In this example, the ML tree (indicated by a red circle) exhibited inaccuracy closely approximating the average for the optimality forest. The existence of numerous phylogenies in the optimality forest may lead different runs of the same program to land on different phylogenies, resulting in computational irreproducibility characterized by different topologies, log-likelihoods, or accuracies. However, the alternative phylogenies inferred due to irreproducibility are likely to have similar accuracies, on average (e.g., figs. 5 and 8).

The presence of an optimality forest suggests that a more extensive heuristic search may not improve the accuracy of the phylogenetic inference. However, more extensive heuristic searches will likely result in more reproducible phylogenies. In fact, 100% computational reproducibility can be achieved through exhaustive searches (or very expansive heuristic searches), which would also yield the ML tree. However, as our findings suggest, the ML tree may not reconstruct the evolutionary relationships more accurately than other trees in the optimality forest. Therefore, improving the reproducibility of the inferred phylogeny for a data set does not necessarily lead to more accurate evolutionary relationships.

**Fig. 9.**
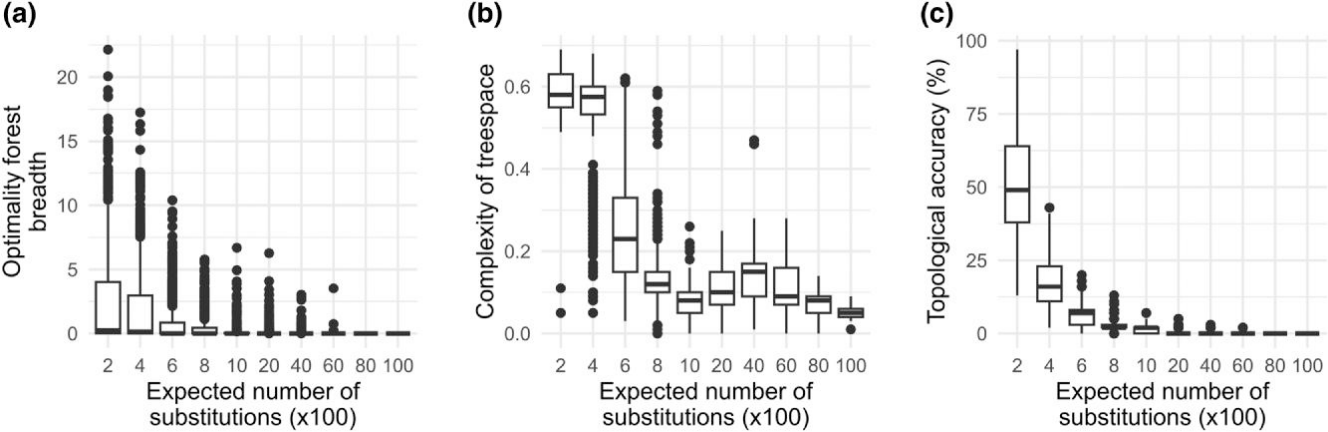
The importance of phylogenetic information on the reproducibility and accuracy of phylogenies is exemplified using the 7,500-data sets collection. The relationship between the number of substitutions contained in an alignment with (*a*) the breadth of the optimality forest, (*b*) the topological complexity of the treespace estimated by Pythia scores, and (*c*) the phylogenetic inference. The expected number of substitutions in an alignment (phylogenetic information) was calculated by multiplying the sum of branch lengths of the true tree and the alignment length.

This association between reproducibility and accuracy observed by Shen et al. (2020) arose because the optimality forest is expected to be bigger for alignments with lower phylogenetic information, measured in the units of the number of substitutions. For example, the breadth of the optimality forest—the difference in log-likelihoods between the true tree and the tree with the highest log-likelihood found—is greater for data sets with fewer substitutions in the 100-data set collection (fig. 9*a*). This breadth will decrease to zero when the number of sites, and thus substitutions, becomes infinity, as the ML method is statistically consistent when all the model assumptions are met. Data sets with less phylogenetic information require more extensive heuristic searches to find the ML tree (fig. 9*b*). When identical heuristic search parameters are employed across all data sets in a collection, some inferred phylogenies become irreproducible for data sets with less phylogenetic information. This results in an artificial correlation between irreproducibility and inaccuracy, as the data sets with less information also yield less accurate phylogenies (fig. 9*c*).

## Conclusions

The computational irreproducibility of phylogenies is a natural consequence of employing heuristic searches for the ML tree. Heuristic searches are necessitated by the fact that the universe of possible trees grows exponentially with the number of sequences (Felsenstein 2004). The widely used software packages use smart algorithms to generate multiple excellent initial trees, which are excellent starting points for heuristic searches. These searches evaluate variations of these initial trees through topological rearrangements and greedy hill-climbing strategies to find trees with higher log-likelihoods (Swofford 1999; Price et al. 2009; Kozlov et al. 2019; Minh et al. 2020; Tamura et al. 2021). This method explores many tree islands and, as we observed, consistently identifies phylogenies with log-likelihoods exceeding those of the true tree (figs. 5 and 7). This implies that the heuristic searches implemented in popular programs are highly efficient in accessing the optimality forest and may achieve accuracies comparable to that of the ML tree. Our results suggest that the lack of computational reproducibility is not a substantial issue in phylogenetics. Still, any negative impacts of irreproducibility on downstream inferences can be mitigated using statistical support metrics (such as bootstrap support values) and presenting consensus phylogenies obtained from multiple runs of heuristic searches with different seeds and tuning parameters (Navidi et al. 1991; Kumar 1996; Morel et al. 2021). In our view, the more significant challenges that molecular phylogenetics confronts are the lack of robustness and the presence of bias because of methodological choices for sequence alignment and tree inference algorithms, the use of different evolutionary models, the selection of genes and genomic segments to be analyzed, as well as the inclusion or exclusion of certain taxa or sequences.

## Materials and Methods

### Simulated Data sets

We used 100 simulated data sets generated in a previous study (Tamura et al. 2012) under an autocorrelated rate model among lineages, as extensive rate correlation has been found in many large empirical data sets (Tao et al. 2019). These data sets were generated under a wide range of sequence lengths (258–9,359 sites), evolutionary rates (0.81–3.95 × 10^−9^ substitutions per site per year), base composition bias (GC% = 39–82%), and transition/transversion rate ratios (1.35–2.6) under the HKY model (Hasegawa et al. 1985). We used subset alignments of 142 mammalian species from the original simulated alignments of 446 vertebrates to reduce the computational burden in ML inferences (fig. 1*a*).

We also re-analyzed a collection of 15 × 500 simulated sequence alignments (7,500-alignments data set) from Shen et al. (2020). Alignments were generated at 15 levels of informativeness, where the average number of parsimony informative sites ranged from 20 to 530. At each level, 500 alignments of 64 taxa with different lengths (300–1,000 sites) were simulated under the GTR + G4 model (gamma rate heterogeneity = 1.0) for modeling a complex evolutionary process. More details of simulation conditions can be found in the original article (Shen et al. 2020).

Finally, we randomly selected 1,000 alignments from the empirical ruminant data set published by Chen et al. (2019). This data set was selected because branch support for both ML and MSC analyses was remarkably high for all nodes. We repeated the analyses for simulated data using a reduced data set including only 14 Bovidae species (fig. 1*c*). Sequence length ranged from 201 to 12,216 bp, and the substitution model used for each alignment was selected by ModelFinder (Kalyaanamoorthy et al. 2017).

### Phylogenetic and Log-Likelihood Differences Between Trees

In the analysis of the 100-alignments collection, we used IQ-TREE 2.1.3 (Minh et al. 2020) and RAxML-NG 1.1.0 (Kozlov et al. 2019) for each data set twice (run 1 and run 2) under the HKY substitution model (matching the simulation conditions) and a log-likelihood epsilon of 0.0001 for optimization. A small epsilon value was used to better optimize the likelihood value in the ML inference and match the analysis conditions used in a previous study (Shen et al. 2020). To ensure consistency, the initial seed of the two runs was fixed to be 111 and 123 for the first and second runs, respectively. Using the same seed in both runs would mandatorily result in the same phylogeny. We compared the log-likelihood values between the trees of two runs and the true tree. The phylogeny of 142 mammalian species for simulating the alignment was used as the true tree for each sequence alignment. We also used the Robinson–Foulds distance (*d*_RF_) to quantify phylogenetic differences between trees and report the percent difference calculated as *d*_RF_/(2 × (*m*−3)) × 100, where *m* is the number of tips.

For the 7,500-alignments collection, the first and second trees produced by IQ-TREE 2 and RAxML-NG and associated metadata were directly retrieved from the supplementary materials in Shen et al. (2020). For a direct comparison, log-likelihoods of the first run IQ-TREE 2 and RAxML-NG trees were re-estimated using the same initial seed used in the original article in IQ-TREE 2. We also compared the topological differences between phylogenies produced in the first and second runs and the phylogeny error of all the inferred trees for each simulated data set. The true tree for each corresponding alignment was the 64-taxa phylogeny used for simulating the alignment. We only discuss results where trees were inferred using IQ-TREE 2 and 2 CPUs. Results from multiple CPUs analyses, star tree simulations, and RaxML runs were qualitatively similar, so they are not presented.

Finally, we evaluated the difficulty in inferring the correct tree from each alignment with Pythia (Haag et al. 2022). The Pythia score evaluates the difficulty of inferring the ML tree based on the complexity of the tree space. We associated this score with the phylogenetic information in each alignment, represented by the total number of substitutions. A small fraction of the alignments had to be excluded from this analysis because Pythia does not calculate scores for alignments containing two identical sequences.

### The Optimality Forest of Trees

We conducted 100 heuristic searches in MEGA-CC (Kumar et al. 2012; Tamura et al. 2021), starting with different initial trees to estimate optimal likelihood trees. These initial trees were produced by the bootstrap procedure in IQ-TREE 2 on all the sequence alignments in the 100-data set collection. Generally, programs do not output intermediate trees, so we modified MEGA-CC such that all the intermediate trees evaluated during the heuristic search were retained. Then, IQ-TREE 2 was used to compute the log-likelihoods of all these intermediate trees to identify trees with optimality scores better than the true tree. Note that the intermediate trees may vary among programs. The width of the optimality forest is the log-likelihood difference between the final inferred tree and the true tree. For all other data sets, the width of the optimality forest was calculated as the difference between the maximum log-likelihoods of the inferred phylogenies and the true tree, which yields an estimate of the minimum width because more heuristic searches with different random seeds and initial trees may produce phylogenies with higher likelihoods.

## Data Availability

All the analyzed data sets and files containing analysis options are available on GitHub (https://github.com/cathyqqtao/Reproducibility).
